# The Effect of STEAM Activities Based on Experiential Learning on Ninth Graders’ Mental Motivation

**DOI:** 10.3390/ejihpe13070091

**Published:** 2023-07-10

**Authors:** Naela Mater, Wajeeh Daher, Fayez Mahamid

**Affiliations:** 1 Department of Ecducational Sciences, An-Najah National University, Nablus P.O. Box 7, Palestinemahamid@najah.edu (F.M.); 2 Department of Mathematics, Al-Qasemi Academic College of Education, Baqa P.O. Box 124, Israel

**Keywords:** STEAM, STEAM activities, mental motivation, experiential learning

## Abstract

The impact of STEAM (Science, Technology, Engineering, Arts, and Math) on pupils’ learning has been increasingly highlighted recently. This study aims to shed light on the effect of STEAM activities based on experiential learning on ninth graders’ mental motivation and learning. The present research adopted a mixed methodology (quantitative and qualitative). The study sample consisted of 90 students divided into three groups. The tools utilized in conducting the study included California Measurement Mental Motivation, and semi-structured interviews with (10) participants. The tools’ validity and reliability were verified. After data were analyzed, the findings showed statically significant differences between students’ post average scores regarding mental motivation due to teaching method, and in favor of the experimental groups (face-to-face STEAM activities, online STEAM activities). This provides tangible proof for the need to include STEAM activities in school curricula to enhance learners’ curiosity, problem-solving skills and self-confidence through learning, as well as their task accomplishment ability.

## 1. Introduction

The STEM (Science, Technology, Engineering, and Math) approach is a contemporary trend for education reform that emerged during the last decade of the last century in the US National Science Foundation (NSF). The United States applied it as an education system radical change and reform due to students’ low achievement rates in national mathematics and science tests [1].

The philosophy of the STEM approach is based on the knowledge unity principle and its functional form, through educational situation designs and activities where the barriers between science, technology, engineering and mathematics disappear [1]. These activities may be consistent with the experiential learning theory proposed by Kolb. It focuses on the principle that learning takes place through concrete and abstract processes. It confirms that the student’s knowledge structure is formed through his/her representation and adaptation processes, and through interaction with their environment [2].

Dark and Burns [3] explained that there were three levels of integration in the STEAM approach: multidisciplinary, in which students learn concepts and skills in each field with reference to the common topic. The second is interdisciplinary, in which they learn principles, concepts and skills from one or more related disciplines. The third is transdisciplinary, in which they tackle real-world problems or projects, apply their knowledge and concepts from two or more areas to help shape their own learning experiences.

Later, the STEM curriculum evolved into STEAM (Science, Technology, Engineering, Art, and Math) by adding Art as a tool for developing creativity and innovation, focusing on skills and processes alongside knowledge [4].

### Research Problem

Land’s study [5] confirmed that recent graduates of STEM majors did have a spirit of innovation and creativity. That is why the USA has sought to adopt the STEAM approach, which is concerned with providing students with high-tech skills, in a way that represents creativity in an artistic form that reduces stress and anxiety, encourages students to explore and increases their internal motivation, and that reflects positively on student learning [6]. 

The COVID-19 pandemic led to the closure of schools and educational institutions, converting educational systems in different countries to distance learning [7]. So, those interested in the STEM approach had to adapt its activities to fit distance learning features. They designed online STEM activities that were suitable for teaching—learning strategies, modeling-simulation programs and other digital applications. 

The rise in online learning and the national focus on STEM education created an urgent need to advance educational best practices that addressed the challenges of delivering high quality online STEM education. Chen et al. [8] recommended that online STEM activities must be designed to be effective and include integrated active learning activities, interactive participation, and appropriate assessment strategies. On the other hand, learning via the Internet means that students are exposed to many types of information from multiple learning sources, dealing with diverse scientific and life problems, in which contradiction is the main feature. This encourages students to practice a set of mental motivation skills such as focusing, learning orientation, creative problem-solving and cognitive integration. It also requires educational institutions to adopt multiple methods to stimulate students’ mental motivational skills to direct them to accomplish tangible innovations and motivate them to accomplish various missions and solve problems in different ways [8].

This study seeks to identify the effect of STEAM activities based on experiential learning on ninth graders’ mental motivation.

## 2. Literature Review

### 2.1. STEAM Approach

The STEAM approach resulted from the STEM approach, as educators saw the additional benefit of adding Art to the Sciences, especially after they saw the advantages of STEM to students’ learning of the Sciences. Daher and Shahbari [9] studied the design of STEM activities by prospective secondary school mathematics teachers, where the findings indicated that those participants found difficulty in designing these different types of activities. Anabousy and Daher [10] studied the design of STEAM activities by prospective elementary school teachers. They found that the designed units took care, primarily, of the individual’s learning of the STEM skill, neglecting the collaborative skills.

Yakman [11] confirmed that the concept of STEAM could take one of two directions: education that includes the integration of STEM disciplines with other fields such as Art, or an integrated education between the disciplines of Science, Technology, Engineering, Art and Mathematics in a way that presents STEAM subjects together. Wynn and Harris [12] indicated that the STEAM approach included many interconnections and exchanges, such as, in the service of art, projects that integrate art into STEM education and projects based on art and STEM equally. The National Art Education Association (NAEA) had stated that STEAM education is “an approach in which art and design principles, concepts, and techniques are integrated into the teaching and learning of science, technology, engineering, and mathematics” [12].

The findings of previous studies revealed the importance of integrating arts with STEM fields to equip students with design skills, stimulating creativity and innovation, and developing critical thinking and problem-solving [3,4,13,14,15,16]. In addition, it played an important role in learning and content retention in art and science [6], contributed to the development of scientific practices among students, increased students’ conceptual understanding [17], motivated students and teachers to become lifelong learners, helped students understand old topics in new ways and refined their personalities through their participating in the learning process, discussion, dialogue, and expressing opinions through working with student groups, as it is compatible for all students, regardless of their differences [18]. This was emphasized in a study by Shahda et al. [19] which stated that one of the goals of the STEAM approach was to take into account all the learning styles and the multiple intelligences of students, to support them, stimulate their senses and social growth, increase their social skills, stimulate their minds, develop their mental thinking skills, link the school curriculum with society and daily life and to prepare them to encounter the changing world challenges. Additionally, Wang et al. [20] indicated that it was necessary to move to STEAM education in order to match the goals of sustainable development with the goals of education in the 21st century.

### 2.2. STEAM Activities Based on Experiential Learning

Kolb believed that learning occurs when the learner understands new experiences and applies them in new situations. That can take place through two orthogonal dimensions, each with two poles: after perception; this consists of Concrete Experience (CE) and ends with Abstract Conceptualization (AC) while, after information processing, it consists of Reflective Observation (RO), ending in Active Experimentation (AE). These are the four dimensions upon which the experiential learning (EL) cycle is based [21]. Parno et al. [22] conducted a study to investigate the effectiveness of an experiential learning (EL)—STEM model with formative assessment (FA) in building fluid dynamics mastery and exploring students’ difficulties. The results showed that the experiential learning—STEM model with formative assessment (FA) increased students’ conceptual mastery.

Smith and Rayfield [23] developed two units of STEM-enhanced instructions, each with two separate sequences; one presented concepts with a concrete experience then moved to abstract conceptualization, and vice versa. The results confirmed that cognitive sequencing could be the most feasible method for educators to follow, as it was compatible with each student’s preferences. 

In this study, a set of STEAM activities were designed based on Kolb’s theory of experiential learning in its four stages, where students worked in groups to carry out practical experiments, such as building an electrical circuit using the available physical components and measuring devices, as a concrete experience. Then, they reflected on the procedures and collected the results in order to obtain the new information. After that they analyzed the results, deduced new concepts and relationships and answered the evaluation questions at the end of each activity. Finally, they moved into the active experimentation phase and applied what they had learned collectively.

### 2.3. Mental Motivation

Educational researchers have been interested in the motivation of students to learn the sciences for a long time; this interest has included the different aspects of motivation, including the following. Firstly, it included students’ motivation in specific contexts. Daher [24] studied students’ motivation to learn mathematics while carrying out modeling activities with technology. He found that, as the group members worked with the internet and GeoGebra to model the Roc bird, they utilized primarily processes-based motivational styles, but did so keeping their primary goal in mind. In addition, researchers studied the relationships between motivational components and other variables. Daher et al. [25] found that each of the three variables—student’s ability in science, their school level and the teacher’s gender—was moderated between student gender and a component of science motivation.

Mental motivation is an important variable that drives a person’s creativity, as it refers to the individual’s internal motives and desires to use creative abilities in the thinking and cognitive processes that can be used in solving problems and making decisions [26]. Other educators have described it as a condition that qualifies a person to accomplish many innovations and solve problems in multiple ways, even those which sometimes seem unfamiliar [27]. It also pushes the individual to integrate and engage in the process of thinking, searching for complex problems and being prepared to find potential solutions, based on searching for reasons and evidence and making the right decisions. Mental motivation contributes to helping students find appropriate solutions to the problems they face by providing various questions that help them in thinking in new ways [28].

De Bono [29] emphasized that the importance of mental motivation was to develop the learners’ ability to make decisions, solve problems in creative ways, push them to self-reliance, take responsibility when completing tasks, and keep them away from external stimuli. Also, it promotes their desire to work and complete tasks and not give up, and to achieve attention and focus when solving problems. In addition, mental motivation also motivated learners to perform higher mental operations and to generate innovative, unfamiliar solutions [30].

## 3. Materials and Methods

### 3.1. Research Design

The study used a mixed method, which combined quantitative and qualitative approaches to obtain a deeper understanding of the studied variables [31]. This methodology was adopted since mental motivation has been little studied using the qualitative method. Since mental motivation was studied mainly quantitatively, we wanted to follow previous studies in doing that, but, at the same time, we wanted to use the qualitative method for two reasons. The first was to gain deeper insight into the phenomenon, while the second was to enable triangulation of the research findings. 

The phenomenology design (or the personal experience of individuals) was adopted for the qualitative approach, to examine the students’ experience upon implementing STEAM activities, and identify the effects of this on students’ mental motivation. Semi-structured interviews were conducted to collect data. This study relied on a quantitative experimental design with pre- and post-measurement, following two methods: 1—teaching students using face-to-face STEAM activities and 2—teaching students using online STEAM activities. Students were divided into 3 groups (control group and two experimental groups) to achieve the purpose of the study.

### 3.2. Research Questions

The study sought to answer the following questions:

- What is the effect of STEAM activities based on experiential learning on the mental motivation of ninth graders?
- How did STEAM activities based on experiential learning affect the mental motivation of ninth graders?

The aim of the first question was to examine the impact of STEAM activities on students’ mental motivation quantitatively, by using collected data statistical analysis. The second question aimed to understand students’ perceptions from their own experience regarding the implementation of STEAM activities in science classes. Allowing students to express their perceptions and tell their stories when carrying out STEAM activities will pave the way for educators to explore the impact of such activities on students’ mental motivation.

### 3.3. Context of STEAM Activities

STEAM activities were applied in science lessons at Nour-Shams School in the Tulkarm District, which is UNRWA-affiliated. The face-to-face and online STEAM activities were designed by the researcher, who was experienced in STEM education. A special teacher’s guide was prepared to help in applying STEAM activities in science classes, in addition to a student’s notebook for face-to-face STEAM activities, in which students carried out the activities and recorded the results and reflections. As for online STEAM activities, the STEAM website was programmed using the C++ language and consisted of short videos, simulation programs, such as virtual lab from PHET website, and digital assessments using Google Drive forms. Students used tablets with stable Wi-Fi during science classes.

#### Example of STEAM Activities: The Electric Current Lesson

The activity concerned the drawing of the amount of charge passing in a specific time in the Cartesian coordinate system. It also requested determining the type of the resulting curve and finding its slope. Such skills (drawing a mathematical relationship through a graph, the Cartesian level, finding the slope) are mathematical skills that the student, in order to understand them, must apply in solving applied problems that are presented in STEAM activities. The students of the experimental group 1 carried out this activity on their notebooks using tangible tools and materials such as copper tape, batteries, a light-emitting diode (LED), etc., and they recorded their observations and reflections, carrying out all scientific inquiries in each activity.

### 3.4. Participants

The study sample consisted of 90 students from a middle school in Nablus, Palestine; none had any previous STEAM experience. They were selected randomly: 30 in the face-to-face STEAM activities experimental 1 group, 30 in the experimental 2 group, in which online STEAM activities were implemented, and 30 in the control group. Ten students from the experimental groups participated voluntarily in the semi-structured interviews (see Table 1). The purpose of the interviews was to explore the effect of STEAM activities on ninth graders’ mental motivation. Face-to-face and online STEAM activities were described, with their impact during science classes. Parental and teachers’ consent were provided. Table 1 includes characteristics of those who participated in the interview, where the names are fictive. 

### 3.5. Data Collection Tools

Several data collection tools were applied, including a survey as well as interviews with the following illustration:

#### 3.5.1. California Measure of Mental Motivation (CM3)

This tool was built by Giancarlo and Facione [30] in order to measure the mental motivation of middle school students. In its original form, the scale consisted of (25) items distributed over four dimensions:

- 6 items for the dimension of Learning Orientation.
- 6 items in the dimension of Creative problem solving.
- 8 items in the dimension of Mental Focus.
- 5 items for the dimension of Cognitive Integration.

The questionnaire was built based on a five-dimensional Likert Scale, with the following weights: 1 degree (strongly disagree), 2 degrees (disagree), 3 degrees (neutral), 4 degrees (agree) and 5 degrees (strongly agree).

The researcher converted the scale into Arabic, revised by translation expert to ensure the veracity of its content. Then, the scale was applied on the exploratory sample consisting of (100) ninth graders, in order to verify its validity and reliability in the Palestinian environment.

The scale’s validity indications were verified by finding out the coefficients of the paragraphs’ correlations with their fields (the sincerity of the internal consistency), followed by finding out the coefficients matrix of the domains’ correlations coefficients with the scale as a whole (see Table 2). The reliability value of the scale was 0.891. The reliability values of each subscale ranged from 0.744 to 0.933.

#### 3.5.2. Semi-Structured Interviews

Data were collected from semi-structured interviews, which were conducted with the aim of exploring students’ current experience regarding face-to-face and online STEAM activities application, and their impact on ninth graders’ mental motivation during science classes. The interview items were based on the research questions, where an interview protocol was developed to guide the interviewers during the interviews. It consisted of eight open-ended questions and a series of probing questions used to extract more in-depth responses from participants who signed a consent form for recording the interviews, which lasted for 30–45 min at the Nour-Shams Girls School. 

### 3.6. Process

All groups were engaged in (20) science classes, over 4 weeks.

- Instruction in the experimental groups

There are two experimental groups in this study; the experimental group (1) consisted of 30 students divided into 6 heterogeneous collaborative working groups taught by face-to-face STEAM activities. They studied the electricity activity unit (from a technology textbook) based on the integration of science and mathematics. The activities consisted of constructing electrical circuits, discovering the relationships between electrical concepts such as voltage, current, and resistance, then drawing them graphically and finding linear equations. These activities were applied in the students’ STEAM notebook, side by side with other small projects. 

The experimental group (2) consisted of 30 students divided into 6 heterogeneous collaborative work groups taught by online STEAM activities individually and collectively. They studied the electricity unit as a digital technology activity based on the integration of science and mathematics. These activities consisted of constructing electrical circuits using the virtual lab, finding out the relationships between electrical concepts such as voltage, current, and resistance by drawing the relationships graphically and looking for linear equations using simulation programs on the PHET website.

- Instruction in the control group

The control group consisted of 30 students who were taught science by traditional strategies. The control group studied the electric current and electrical resistance concepts by providing definitions, examples and applying the curriculum activities.

### 3.7. Data Analysis

- Analysis of qualitative data

The audio files’ content was analyzed based on the procedures of Yin [32]. We followed the analysis of previous qualitative studies [33,34]. Similar responses were selected to be units for text analysis. One of the basic principles was to analyze results sequentially or iteratively, by focusing on data subsets, to obtain more sensitive results [35], which could be a single word, a sentence or several phrases. Data saturation was reached after analyzing the interviews of eight students, but the analysis continued to ensure that all the related categories and properties of the categories were arrived at [36].

- Analysis of quantitative data

Covariance analysis (ANCOVA) was conducted to analyze the effects of STEAM activities on students’ mental motivation. ANCOVA evaluates whether the population means of a dependent variable are equal across levels of a categorical independent variable by statistically controlling the effects of other continuous variables that are not of primary interest, known as covariates [35]. In this study, face-to-face and online STEAM activities were the independent variables and the students’ mental motivation was the dependent one; the scores of the pre-test were described as “covariate” variables.

## 4. Results

### 4.1. Mental Motivation

The descriptive statistics of the CM3 pre-test and post-test are illustrated in Table 3. The results in Table 1 show that there was an increase in the post-test mean scores of the students’ mental motivation in both experimental groups.

The results in Table 4 showed that there was a significant difference between the experimental groups and control group in terms of their mental motivation at the end of the application in favor of the experimental groups, (F (2,86) = 23.909, *p* = 0.000, Partial µ2 = 0.357). Moreover, the effect size was large. The findings indicated that the differences in the mean scores of the students in the post-test of CM3 were attributed to teaching method.

The adjusted means of the groups were calculated in Table 5, to find out the direction of the differences in the average performance of the study groups (the control who studied in the traditional way, or the experimental group (1), who were taught using face-to-face STEAM activities, and the experimental group (2), who were taught using online STEAM activities).

The results of Table 4 showed that the difference in the mean between the study groups was in favor of the experimental groups that were taught using face-to-face and online STEAM activities; the adjusted means of the experimental group (1) and the experimental group (2), respectively, were 3.820 and 3.910, which were greater than the adjusted mean of the control group (3.429) in the CM3. To examine the statistical significance of these differences between groups, pairwise comparisons were performed using the LSD test to make comparisons between the means of the three methods (see Table 6).

The results indicated that there was a statistical significance between the mean scores of the experimental group (1) and the control group in favor of the experimental group (1), in addition to a significant statistical significance between the mean scores of the experimental group (2) and the control group, in favor of the experimental group (2). So, the findings indicated that the use of face-to face and online STEAM activities were more effective in improving the mental motivation of students than traditional teaching methods.

### 4.2. Interviews Results

The participants expressed their pleasure in studying through face-to-face and online STEAM activities; they considered this a special learning experience. To answer the second research question, the researcher conducted a triangulation analysis between the data sources. The semi-structured interviews and CM3 and the findings were categorized into four main themes: Learning Orientation, Creative Problem-Solving, Mental Focus and Cognitive Integration. Each main theme contained a group of sub-themes that the researcher derived from the interviews. 

#### 4.2.1. Learning Orientation

Most of the interviewees reported that the application of face-to-face and online STEAM activities had a positive impact on mental motivation in the field of orientation towards learning regarding several aspects that formed the following sub-categories:

- Desire to engage in new experiences

Most of the interviewees reported that the STEAM activities aroused their curiosity and eagerness to engage in new experiences. Salma said: “I enjoy learning new things, my mind opened up, and it absorbed more things, it came to my mind that I learn new things, I was very happy, and I felt comfortable, I never got tired of learning something new”. Another participant in the online STEAM activities group (Noor) said: “Yes, I loved learning new things, but sometimes I got tired of it, and sometimes I faced some difficulty, but it was not a big issue because I liked the path of knowledge and learning new things that may benefit my life”. 

We notice that face-to-face and online STEAM activities promoted students’ motivation towards gaining new knowledge and searching for information from multiple sources.

- The importance of learning

The interviewees indicated that STEAM activities helped them realize the importance of learning, as it expanded knowledge through exploration, promoting their involvement in the learning process and tackling difficult tasks, Ruba said:

*“Yes, it was very important. I mean, every time I searched for information and got it by myself without referring to someone else, I would benefit more from it in my life and I could teach it to others. It was very sweet, and I would always be happy about it”.*

Sara considered learning as their future, which constitutes an added value:

*“Learning is my future. My future means education if I study medicine in the future, for example, it will help me. It will also be a sweet thing, which means that I reach the goal in my learning. It was a nice experience that I am very proud of the thing that I had produced. Education and learning are simply our whole life. A person without learning is nothing, frankly”.*

Through the students’ interviews, we note that face-to-face and online STEAM activities increased students’ motivation and engagement in learning.

- Psychological stability

Through the interviews, it was clear that orientation towards learning made students feel psychologically comfortable, happy and like they were having fun. This was concluded from the interviewees’ answers, where Lama said: “When I learn something new, it is something sweet. It means that I reached my goal, a sweet experience. I am very proud of the thing that I produced”. Another participant, Nagham, also said: “Regardless that the activities were easy or difficult, they were very enjoyable, because they were integrated with each other in a way that we could have employed the scientific concepts that we had learned so far”.

It’s obvious that, after the students participated in face-to-face and online STEAM activities, they felt psychological comfort, stability and balance.

#### 4.2.2. Mental Focus

Most of the interviewees reported that face-to-face and online STEAM activities improved their mental motivation by increasing their ability to organize and accomplish tasks.

- Ability to organize a plan to complete tasks

The application of STEAM activities increased students’ ability to be organized with intellectual clarity in implementing tasks. They felt comfortable and self-confidant in being able to complete the required tasks in a timely manner. This was evident when the students were asked about how homework was organized and they all confirmed their keenness to put together an action plan to complete the assignments and even double the needed time, Nuha said:

*“I arranged an appropriate time plan, for example, if I had a math homework that needed half an hour, I devoted a whole hour in the plan, and if I had science homework that consumed an hour, I devoted two hours in the plan. This is how I increased the time for each task to catch up on time and be comfortable”.*

- Problem-solving ability

Most of the interviewees reported that STEAM activities increased the students’ ability to solve problems they had encountered during the implementation of the activities in and with creative, sequential and interconnected ways and ideas.

- Follow the steps in solving problems

The students reported that following the scientific steps in solving problems helped them in facing many difficulties and in thinking scientifically and logically; Mays said:

*“For example, we faced a problem that after completing the installation of the electrical circuit it did not work, so I disassembled the circuit and re-installed it again in order to determine the flaw which may occur in the direction of the electrodes, the type of wires, or the battery, so I started collecting data, verifying, and setting hypotheses and proposed solutions to solve the problem”.*

Through the above, it seems that the students encountered problems while carrying out the STEAM activities, but that they solved them logically, which increased their ability to think scientifically and systematically.

- Confront difficulties in solving problems

Some students reported that they felt uneasy and nervous when facing problems, but were happy when solving them. When asked how to tackle difficult problems, Noor from the online STEAM group said:

*“When I faced a difficult problem, at first I felt that it couldn’t be solved. After that I talked to myself, I wanted to see why this happened, so I started making assumptions and searching for reasons. This helped me find the solution, I felt that very happy doing that by myself, learning it all alone”.*

Through the participant’s statement, it is obvious that the implementation of face-to-face and online STEAM activities caused a disturbance in their feelings, but being able to solve the problems without any external support, made them satisfied.

- Insistence on solving difficult problems

The students encountered difficult problems during the implementation of the activities, which made them more determined to solve them and keep trying. Nuha said:

*“There faced some difficulties, for example, when the measurements were scribbled, there were errors, so we repeated the measurements more several times”.*

- Innovation and creativity

The results of the interviews showed that STEM activities encouraged students to choose difficult problems and tasks, and highlight their creativity through innovative projects. Sara said:

*“At the beginning, I chose a task that I could do easily, and learned how to apply it in the difficult ones. I know the basic things I need to learn. But then of course I chose the difficult one in order to create and produce something new”.*

#### 4.2.3. Collaboration with Partners

The students’ encounters with difficult problems, and their inability to solve them on their own, led them to seek cooperation with their colleagues, or to turn to the teacher, an expert or various learning resources. Salam said:

*“When we faced a difficult concept or problem, we were nervous, but then we cooperated with each other in our group, and it was amazing, and if we did not understand, we asked the teacher. We would also resort to YouTube to search for information”.*

#### 4.2.4. Cognitive Integration

- Connecting previous experiences

Face-to-face and online STEAM activities increased the students’ capability of using neutral thinking skills, meaning they could use all the knowledge they possess to solve problems. Ruba said:

*“I used what I had learned in situations that I experienced, for example in the subject of health, we learned how to do first aid and deal with some sick cases. If someone was injured, I know what to do to help”.*

And May reported:

*“When we carried out the activities, it was about finding the slope of a straight line and using the Cartesian level. This was learned in mathematics and applied it in STEM activities in the science class. I understood it in mathematics, but applying it in STEM activities had enhanced my understanding, and I became more aware of its importance and why we should learn it”*

From the students participating in face-to-face and online STEAM activities, we can see that these activities provided educational situations where knowledge that students had in STEAM fields was integrated.

- Caring about what other people think

Participants also reported that they considered other points of view, felt comfortable while carrying out the educational task and enjoyed thinking through interacting with others’ various views. This was represented in the students’ responses about their role when their families faced a specific problem. Sara answered:

*“I cared about my family’s opinion, I shared with them their problems, and we tried together to come up with solutions. For example, I calculated how much electrical energy each device consumed, and determined which device has a high consumption, so that we could reduce operating it as much as possible”.*

Through the students’ reports, we conclude that the STEAM activities helped them to pay attention to others’ opinions and to actively participate in new ideas to reach appropriate solutions.

### 4.3. Considering the Two Types of Results

The statistical results of the quantitative data indicated that the face-to-face and online STEAM activities have a positive effect on the mental motivation, in all its components, among ninth grade female students. To explore qualitatively the effect of these activities on the students’ mental motivation, semi-structured interviews were conducted with 10 female students who carried out face-to-face and online STEAM activities. After collecting, unpacking, coding and analyzing the data, a group of main themes appeared, which agreed with the four areas of mental motivation: learning orientation, mental focus, creative problem-solving, and cognitive integration. We concluded that the quantitative and qualitative results agreed regarding the positive impact of STEM activities on students’ mental motivation. For example, the qualitative results indicated the importance of STEAM learning for the students, as it is very important and shapes their future and the learning process creates psychological stability for the female students and makes them feel psychologically comfortable, happy and like they are having fun.

In addition to the above, the students expressed an increase in their ability to face problems and their orientation to solve them creatively by following the steps of solving the scientific problem while cooperating with their colleagues. Moreover, the female students confirmed that face-to-face and online STEAM activities increased their ability to accumulate previous experiences, so they could use all the knowledge they possess to solve the problems they faced. 

## 5. Discussion

STEM and STEAM education are attracting the attention of educators and researchers [37,38,39]. In this paper, the effectiveness of STEAM activities on the mental motivation of ninth graders in science classes was examined, addressing both face-to-face and online STEAM activities. The results showed the superiority of face-to-face activities over traditional teaching methods in developing mental motivation from several aspects.

The research results indicate that the face-to- face STEAM activities helped motivate students, enhance their interest in learning and push them to perform the required educational tasks and come up with creative ideas. This, in turn, helped develop the students’ mental motivation in the field of Learning Orientation. These results agree with previous studies [2,19,20] that indicated that the application of STEM activities based on experiential learning enabled students to gain useful educational experience, build deep concept mastery, reflect, draw conclusions, and develop collective work skills to apply their gained knowledge to solve daily issues.

Face-to-face STEAM activities stimulated the students’ minds and directed their mental behavior towards solving problems, especially when they faced a problem, for example, the failure of an electrical circuit due to a defect in the connection, where they worked on rebuilding the circuit several times, which increased their perseverance for fulfilling their goal, deducing the results and then writing the report and answering the questions in the student’s notebook. This, in turn, helped develop their skills in solving problems following the scientific methodology, and increased their determination to face difficult problems and develop appropriate solutions. This result agrees with other studies which explained that placing students in educational situations that include realistic problems motivated students to think and pushed them to solve problems, which increased their mental motivation [4,37]. In addition, teamwork and collaboration helped them to develop social skills and build relationships with their peers. This is consistent with previous research [38,40], which emphasized the role of student robot projects in developing social skills among students.

These activities helped to develop the students’ mental thinking skills and the ability to organize their work, by dividing the task into small sub-missions that are completed sequentially. This means completing the main task, and connecting the STEAM activities with social life in order to solve daily problems, which constitutes the Mental Focus aspect of mental motivation. This interpretation agreed with related studies [16,19], which emphasized that the integration and linking of topics helped students understand difficult scientific concepts.

Moreover, the integrative activities were also designed in a way that helped students understand pre-learned topics in mathematics in an integrative manner with science topics, such as in finding mathematical relationships between electric current, voltage, and resistance, which are physical variables, in graphing on the Cartesian plane and in finding the equation of a straight line. Furthermore, these activities helped students to engage in difficult and ambiguous tasks, which required investigation, extrapolation, conclusions, reflection, and to understand the multiple points of view in an impartial manner through discussions and dialogues that took place within work groups. This, in turn, helped develop the students’ mental motivation in the field of Cognitive Integration, something which agrees with previous studies [10,37] in showing the importance of integrating STEAM topics to motivate students, increase their understanding of knowledge and concepts and help them solve problems.

Similarly, the results also showed the superiority of online STEAM activities over the traditional teaching method in developing students’ mental motivation in all fields. The researcher believes that these activities provided students with good opportunities for learning and orientation towards discovering new knowledge through the implementation of individual digital projects represented in watching short educational videos, conducting experiments using the virtual laboratory (PHET), and using virtual digital electronic devices to take measurements and record them in tables designed to extrapolate the results. This is consistent with the results of the studies of references [5,39,40] that emphasized the role of online STEM activities in improving students’ access to information, increasing their domestic learning opportunities.

The attachment of an electronic formative evaluation after each activity helped students to preserve the impact of learning and organize their thoughts and tasks, added an atmosphere of fun and contributed to the development of skills such as problem-solving, data access and analysis, creativity and critical thinking. This result agrees with the study of McNamara et al. [14], which confirmed that interactive online STEM activities increase students’ ability to think critically, and the use of such activities can be easily managed online in the classroom and beyond and can also be manipulated to fit multiple topics within STEM fields. The result also agrees with the study of Ozdemir [7], which indicated that online STEM activities are fun and interesting and that they are useful for students to maintain knowledge, even though they can be difficult and complex. The same is true for Ransdell’s study [41], which confirmed that the students’ integration into digital activities via the Internet increased their ability to organize their thoughts and tasks and this, in turn, increased their mental focus, which is one of the areas of mental motivation.

The online module and tablets were provided to motivate and attract students’ interest in learning (orientation towards learning), and encourage them to carry out continuous research processes and generate creative ideas. Linking information, such as with mathematics (the equation of a straight line and slope), and representing the data graphically to find relationships between them through the PHET website, with newly learned scientific concepts, gave students the opportunity to learn better and to appreciate the importance of what they learn in life. This interpretation agreed with the results of the studies in references [5,42,43,44], which indicated that online educational courses and activities increased the chances of students’ learning success.

## 6. Conclusions and Limitations

### 6.1. Enriching the Curricula and Textbooks with STEAM Activities to Ensure Mental Motivation

The results showed that the application of face-to-face STEAM activities increased the students’ mental motivation level; therefore, the researchers recommend that male and female teachers consider the development of mental motivation among students to increase their desire and tendencies towards learning, deal with problems and promote their ability to integrate knowledge through the use of various teaching strategies that seek to develop mental motivation and provide the appropriate classroom environment for this purpose. Based on the above, the researchers suggest that decision makers enrich the curricula and textbooks with STEAM activities to ensure the achievement of cognitive integration, and that they train science teachers to employ the STEAM approach within classes, in addition to expanding the current study, taking into account the variables of gender and other school grades. 

### 6.2. Enriching the Curricula and Textbooks with Digital STEAM Activities to Ensure Mental Motivation

The results also showed that the application of STEAM activities via the Internet increased students’ mental motivation level. Therefore, the researchers recommend that male and female teachers need to use digital simulation programs and electronic student learning assessment sites, such as Kahoot and Google Form, and build electronic courses, that integrate students’ knowledge in science and mathematics and provide applied situations in which students practice what they have learned so far, in a way that develops their motivation towards learning and increases their ability to learn and master knowledge. Accordingly, the researchers suggest conducting more studies based on the development of e-learning units according to the STEAM approach.

### 6.3. Limitations of the Study

The main limitation of the experimental result was the relatively small sample size, as it was from one school in the Nablus educational area for the ninth grade, and was limited to the female category only, which limited the generalizability of the results. In future studies, it is possible to evaluate the effect of STEAM activities on a larger sample size in multiple geographical areas for both males and females. Also, the students’ academic abilities were not taken into account in this study, so it is possible to conduct future studies examining the impact of STEAM activities on students of different academic abilities. It will be useful to conduct prospective studies examining the impact of STEAM activities on different school grades.

## Figures and Tables

**Table 1:** Participants in semi-structured interviews.

Group	N	Nick Name	Age
Experimental 1 (Face-to-Face STEAM activities)	5	Salma	15
		Lama	15
		Ruba	16
		Mays	15
		Nuha	16
Experimental 2 (Online STEAM activities)	5	Rasha	16
		May	15
		Nagham	15
		Noor	15
		Sara	15

**Table 2:** The values of the correlation coefficients between the score of each item and the total score of the domain.

Domain	Paragraph	Item Coefficient	Domain Coefficient
Learning Orientation	I love learning new things.	0.738 **	0.648 **
	I always look forward to learning challenging things.	0.910 **	
	Being eager to learn about different things is one of my strong points.	0.769 **	
	No matter the topic, I am eager to know more about it.	0.786 **	
	Learning new things all my life would be fun.	0.714 **	
	I want to learn everything I can because it might come in handy someday.	0.594 **	
Creative problem solving	Complicated problems are fun to try to figure out.	0.604 **	0.708 **
	If given a choice, I would pick a challenging activity over an easy one.	0.717 **	
	I really enjoy trying to figure out how things work.	0.668 **	
	Easy problems are less fun than challenging problems.	0.612 **	
	I hate dealing with anything that is complicated.	0.658 **	
	I am good at making plans for how to solve difficult problems.	0.761 **	
	I am one of the smartest kids in my class.	0.663 **	
Mental Focus	I have trouble concentrating in school.	0.856 **	0.790 **
	My trouble is I stop paying attention too soon.	0.871 **	
	It is easy for me to stay focused when working on a problem.	0.836 **	
	It is difficult for me to finish my school assignments.	0.850 **	
	I keep my schoolwork organized.	0.846 **	
	It is easy for me to organize my thoughts.	0.822 **	
	When I need to solve a problem, I have difficulty knowing where to begin.	0.839 **	
Cognitive Integration	It is just not that important to keep trying to solve difficult problems.	0.803 **	0.607 **
	I only look for facts that support my beliefs, not for facts that disagree.	0.687 **	
	Thinking about other points of view is a waste of time.	0.649 **	
	I know what I think, so why should I pretend to consider choices.	0.673 **	
	Thinking about what others believe means you cannot think for yourself.	0.710 **	

** *p* < 0.01.

**Table 3:** Statistics for the pre-test and post-test for CM3.

		Pre-Test		Post-Test	
Group	N	Mean	S.D	Mean	S.D
Control	30	3.505	0.374	3.324	0.274
Experimental 1	30	3.501	0.457	3.911	0.291
Experimental 2	30	3.505	0.374	3.814	0.351

**Table 4:** ANCOVA results for the effects of STEAM activities on mental motivation.

Source	Sum of Squares	df	Mean Squares	F	*p*	µ2
Group	3.918	2	1.959	23.909	0.000 *	0.357
Error	7.047	86	0.82			
Total	1259.326	90				

* *p* < 0.05.

**Table 5:** Adjusted means and S.D for the performance of the study groups in the CM3 according to teaching method.

Group	Adjusted Means	S.D
Control	3.429	0.052
Experimental 1	3.910	0.052
Experimental 2	3.820	0.052

**Table 6:** Pairwise comparisons (LSD-test) results for the statistical differences between the means of the CM3 due to the teaching method.

Group	Other Groups	Mean Difference	*p*
Control	Experimental 1	−0.481 *	0.000
	Experimental 2	−0.391 *	0.000
Experimental 1	Experimental 2	0.090	0.227
	Control	0.481 *	0.000
Experimental 2	Experimental 1	−0.090	0.227
	Control	0.391 *	0.000

* *p* < 0.05.

## Data Availability

The data presented in this study are available upon request from the corresponding author. The data are not publicly available due to privacy concerns.
